# Cystacin C-based eGFR and Creatinine Clearance-based eGFR in Patients with Severe Motor and Intellectual Disabilities

**DOI:** 10.31662/jmaj.2023-0137

**Published:** 2023-10-04

**Authors:** Kouichi Tamura, Toshikazu Takizawa, Gaku Shimura, Toshiharu Kokuho

**Affiliations:** 1 Department of Medical Science and Cardiorenal Medicine, Yokohama City University Graduate School of Medicine, Yokohama, Japan; 2 Department of Renal Medicine, Hadano Red Cross Hospital, Hadano, Japan; 3 Department of Nephrology, Yokosuka General Uwamachi Hospital, Yokosuka, Japan; 4 Department of Renal Medicine, Yokosuka City Hospital, Yokosuka, Japan

**Keywords:** Chronic kidney disease, Kidney function, Cystatin C

Chronic kidney disease (CKD) is a clinical diagnostic definition that comprehensively expresses pathological conditions where kidney damage or decreased kidney function persists ^(1), (2)^. Accumulated evidence has shown that CKD increases the risk of cardiovascular diseases (CVD) such as myocardial infarction, stroke, and heart failure (HF), as well as death (cardiorenal linkage), and CKD should be an important target for primary CVD prevention such as heart failure ^(3), (4), (5), (6)^.

Recent large-scale analysis of a Japanese real-world hospital database has shown that the presence of CKD or HF, in addition to type 2 diabetes, significantly increased mortality ^(7), (8)^. In addition, a large-scale cohort study of patients with CKD in Japan has reported that HF is more frequent than myocardial infarction and cerebrovascular disease as complications among patients with CKD ^(3), (4)^; complications with a risk of acute kidney injury and cardiovascular-kidney comorbidity (i.e., cardiorenal syndrome with HF and CKD) are recent pathological features ^(6)^.

Therefore, the report of the study group on Measures against Kidney Disease, which was created in close cooperation with academic societies such as the Japanese Society of Nephrology, Japanese Circulation Society, Japan Diabetes Society, Japan Medical Association, local governments, and patient groups, was published by Health, Labor, and Welfare Association in July 2018 ^(9)^. It is issued by the Ministry, encouraging the conduction of awareness-raising activities regarding the importance of early detection and treatment of CKD ^(9)^.

Moreover, the number of Japanese patients with CKD is estimated to be approximately 13.3 million, of which approximately 1 in 8 adults has CKD, and the prevalence of CKD is high among the elderly ^(2)^. Subsequently, the aging of patients with chronic dialysis has been noticeable in recent years, and the statistical analysis survey results by the Japanese Society for Dialysis Medicine [the annual report of The Japanese Society for Dialysis Therapy (JSDT) Renal Data Registry (JRDR)] also revealed that the number of patients with chronic dialysis would reach approximately 350,000 by the end of 2021, with an increasing trend in patients aged ≥70 years ^(9), (10), (11)^. Particularly, patients undergoing chronic dialysis are reported to have high mortality and hospitalization rates. Functional dependence is clinically recognized as a contributing factor to subsequent disability, recurrent hospitalization, and increased mortality ^(12)^. An increasing burden of functional dependence has been observed in several studies with progressive deterioration of kidney function; functional dependence may contribute to morbidity and mortality in patients with chronic dialysis, suggesting a problem from the socio-medical viewpoint in Japan and other countries ^(13)^.

Although CKD often presents a few subjective symptoms, it can be easily diagnosed by blood and urine tests. Therefore, the prevention of severity and suppression of CVD onset through early diagnosis, via health checkups, tests at medical institutions, and providing appropriate treatment, is important ^(2)^. Kidney function is assessed using glomerular filtration rate (GFR) and is calculated using the Japanese GFR estimation formula (eGFR-Cr) from serum creatinine level, gender, and age in daily clinical practice^(14)^; additionally, the Japanese GFR estimation formula (eGFR-cysC) based on serum cystatin C levels can be used ^(15)^. If more accurate renal function is required, measured GFR is used using inulin clearance. Creatinine is produced by muscle, and serum creatinine levels are affected by muscle mass, so eGFR-Cr is estimated to be high in patients with decreased muscle mass due to sarcopenia (long-term bed rest, etc.) in elderly people, muscle disease, or limb loss ^(1), (2)^. Conversely, eGFR-Cr is estimated to be low in cases such as athletes who have a habit of exercising and a large amount of muscle mass ^(1), (2)^. Serum creatinine levels are also affected by diet, exercise, and renal tubular secretion ^(1), (2)^. Cystatin C is produced by cells throughout the body, and serum cystatin C levels are not affected by muscle mass but are influenced by thyroid function, smoking, inflammation, fat mass, pregnancy, and immunosuppressants．

Calculating eGFR in patients with severe motor and intellectual disabilities (SMID) using serum creatinine overestimates kidney function due to low muscle mass. Thus, in this issue of JMA Journal, Uemura O et al aimed to evaluate and compare the efficacy of eGFR formulae in patients with SMID by evaluating the efficacy of three eGFR equations, creatinine clearance (Ccr)-based eGFR (eGFR-Cr), serum cystacin C (cysC)-based eGFR (eGFR-cysC), and serum beta-2 microglobulin (β2MG)-based eGFR (eGFR-β2MG), in 18 patients with SMID ^(16)^. Uemura et al described the accuracy of eGFR-cysC, when compared with eGFR-Cr, in patients with SMID. In their study, the authors measured 24h-Ccr in 18 patients with SMID and converted it into inulin clearance (Cin), which is the gold standard for measuring GFR, by an equation developed from children ^(16)^. The authors reported that eGFR-Cr does not reliably estimate kidney function in adult patients with SMID and alternatively recommended using cystatin C (cysC)-based eGFR (eGFR-cysC) in the Japanese population of severe motor and intellectual disabilities ^(16)^. This study was well performed and the results were important and interesting, especially for practitioners and researchers in the fields of kidney medicine and gerontology ^(16)^.

## Article Information

### Conflicts of Interest

None

### Disclaimer

Kouichi Tamura is one of the Editors of JMA Journal and on the journal’s Editorial Staff. He was not involved in the editorial evaluation or decision to accept this article for publication at all.
